# BMC Medical Education reviewer acknowledgement 2015

**DOI:** 10.1186/s12909-016-0573-9

**Published:** 2016-02-09

**Authors:** Clare Partridge

**Affiliations:** BioMed Central, Floor 6, 236 Gray’s Inn Road, London, WC1X 8HB UK

## Abstract

The editors of BMC Medical Education would like to thank all our reviewers who have contributed to the journal in Volume 15 (2015).

**Aase Aamland**

Norway

**Rajib Acharya**

India

**Wilco Achterberg**

Netherlands

**Michelle Aebersold**

USA

**Akisibadek Afoko**

Ghana

**Nelia Afonso**

USA

**Pooja Agarwal**

USA

**Rola Ajjawi**

UK

**Fares Alahdab**

USA

**Najlaa Alamoudi**

Saudi Arabia

**John Albarran**

UK

**Mohamed Al-Eraky**

Saudi Arabia

**Heather Alexander**

Australia

**Carla Alexander**

USA

**Syeda Ali**

Pakistan

**Hanan Al-Kadri**

Saudi Arabia

**Jill Allison**

Canada

**Patricia Alpert**

USA

**Abdullah Alzahem**

Saudi Arabia

**Anthony Amalba**

Ghana

**Santushi Amarasuriya**

Sri Lanka

**Gowri Anandarajah**

USA

**Douglas Ander**

USA

**Liz Anderson**

UK

**Geoffrey Anderson**

USA

**Don Anderson**

USA

**Vivienne Anderson**

New Zealand

**Allen Andrade**

USA

**Matthias Angstwurm**

Germany

**Nigel Armfield**

Australia

**Richard Arnett**

Ireland

**Andleeb Arshad**

Kuwait

**Elpida Artemiou**

Saint Kitts And Nevis

**Aimee Aubeeluck**

UK

**Marc Auerbach**

USA

**Myo Nyein Aung**

Thailand

**Samy Azer**

Saudi Arabia

**Maria Manuel Azevedo**

Portugal

**Warwick Bagg**

New Zealand

**Laila Bahammam**

Saudi Arabia

**David Bahner**

USA

**Yun Bai**

China

**Lubna Baig**

Pakistan

**Paul Baker**

UK

**Constance Baldwin**

USA

**I Bank**

Netherlands

**Carlos Baptista**

USA

**Rebecca Baranowski**

USA

**Judith Barbaro-Brown**

UK

**Patrick Barlow**

USA

**Stephen Barnett**

Australia

**Deborah Barr**

USA

**Tony Barrett**

New Zealand

**Emma Bartle**

Australia

**Ankur Barua**

Nepal

**Joanna Bates**

Canada

**Zeynep Baykan**

Turkey

**Margaret Bearman**

Australia

**David Bearn**

UK

**Stefanie Beck**

Germany

**Gary Beck**

USA

**Jan C Becker**

Germany

**Pierrick Bedouch**

France

**Deborah Begoray**

Canada

**Anne Belcher**

USA

**Mary Bell**

Canada

**Jochanan Benbassat**

Israel

**Deirdre Bennett**

Ireland

**Anne-Marie Bergh**

South Africa

**Esther Bergman**

Netherlands

**Robert Bernard**

Canada

**Allan Bernardo**

Macao

**Cheri Bethune**

Canada

**Deepa Bhat**

India

**Ali Bikmoradi**

Iran

**Rex Billington**

New Zealand

**Justin Bilszta**

Australia

**Adelaide Blavier**

Belgium

**Julia Blitz**

South Africa

**Karin Blomberg**

Sweden

**Daniel Blumenthal**

USA

**Phil Blyth**

New Zealand

**Sylvain Boet**

Canada

**Valdes Bollela**

Brazil

**Katrien Bombeke**

Belgium

**Eveline Booij**

Netherlands

**Thomas Booth**

UK

**Guido Bortoluzzi**

Italy

**Hans Martin Bosse**

Germany

**Joseph Donald Boudreau**

Canada

**Frances Bourbonnais**

Canada

**Malcolm Boyle**

Australia

**Marek Brabec**

Czech Republic

**Carlos Brailovsky**

Canada

**Andrei Brateanu**

USA

**Victoria Brazil**

Australia

**Jennifer Breckler**

USA

**Jan Breckwoldt**

Switzerland

**Gustavo Bregagnollo**

Brazil

**Jarle Breivik**

Norway

**Mette Brekke**

Norway

**David Brinkman**

Netherlands

**Eileen Britt**

New Zealand

**Pier Bryden**

Canada

**Ryan Brydges**

Canada

**Alison Bullock**

UK

**S Bunton**

USA

**Bryan Burford**

UK

**M Burge**

USA

**Annette Burgess**

Australia

**Julian Burton**

UK

**Jamiu Busari**

Netherlands

**Lillian Butungi Niwagaba**

USA

**Carma Bylund**

Qatar

**Brian Cameron**

Canada

**Craig Campbell**

Canada

**Kendall Campbell**

USA

**Angela Carberry**

Australia

**Marian Carey**

UK

**Jan Carline**

USA

**Elisabeth Carlson**

Sweden

**Rosemary Caron**

USA

**Sandra Carr**

Australia

**Enrique Castro-Sánchez**

UK

**Peter Cathro**

Australia

**Bernard Cerutti**

Switzerland

**Wen-Lin Chai**

Malaysia

**Wai Hong Chan**

UK

**Madawa Chandratilake**

Sri Lanka

**Shine Chang**

USA

**Serena Chao**

USA

**Renata Chapman**

Australia

**Rodger Charlton**

UK

**Herbert Chase**

USA

**Julie Chen**

Hong Kong

**Yen-Yuan Chen**

Taiwan

**Jack Chen**

USA

**Yan Chen**

New Zealand

**Han-Sun Chiang**

Taiwan

**Tudor Chinnah**

UK

**Tobias Chirwa**

South Africa

**Margaret Chisolm**

USA

**Yen-Lin Chiu**

Taiwan

**Jane Chueh**

USA

**Wanicha Chuenkongkaew**

Thailand

**Anna Chur-Hansen**

Australia

**Giancarlo Cicolini**

Italy

**Jeannie Cimiotti**

USA

**Sarah Clark**

USA

**Kevin Clauson**

USA

**Jennifer Cleland**

UK

**Lynn Clouder**

UK

**David Coady**

UK

**Deborah Cohen**

UK

**James Colbert**

USA

**Hoffie Conradie**

South Africa

**Ronan Conroy**

Ireland

**Siobhan Cooke**

UK

**Simon Cooper**

Australia

**Marie Corkery**

USA

**Manuel Costa**

Portugal

**Ian Couper**

South Africa

**Alison Cowley**

UK

**Gregory Crawford**

Australia

**Caroline Crawford**

USA

**Hilary Creed-Kanashiro**

Peru

**Jim Crossley**

UK

**Sylvia Cruess**

Canada

**Laura Cullen**

USA

**Sally Curtis**

UK

**Fiona Curtis**

UK

**Tara Cusack**

Ireland

**Eugene Custers**

Netherlands

**Michele Daly**

Australia

**Kay Daniels**

USA

**Mary Dankbaar**

Netherlands

**Jeanette Dawa**

Kenya

**Andrew Day**

Australia

**AJ de Beaufort**

Netherlands

**Walter De Caro**

Italy

**Elizabeth Dean**

Canada

**Clare Delany**

Australia

**Dianne Delva**

Canada

**Marcel D'Eon**

Canada

**Luca Dessy**

Italy

**Shafik Dharamsi**

Canada

**Joost Dijkstra**

Netherlands

**Konstantinos Dimitriadis**

Germany

**Janine Dizon**

Australia

**Mamuka Djibuti**

Georgia

**Sarah Dobson**

Canada

**Eva Doherty**

Ireland

**Anthony Donato**

USA

**Chaoyan Dong**

Singapore

**Peter Donkor**

Ghana

**Lachlan Doughney**

Australia

**Athanassios Douzenis**

Greece

**Alan Dow**

USA

**John Roger Downie**

UK

**Aoife Doyle**

UK

**Brian Drolet**

USA

**Robbert Duvivier**

Netherlands

**Craig Eberson**

USA

**Samuel Edelbring**

Sweden

**Gudrun Edgren**

Sweden

**Daniel Edwards**

Australia

**Jan Ehlers**

Germany

**Diann Eley**

Australia

**Rodrigo Elizondo Omaña**

Mexico

**Simone Elliott**

Australia

**Matthew Ellis**

UK

**Amal El-Moamly**

Egypt

**Adel Elmoselhi**

United Arab Emirates

**Ruth Endacott**

UK

**Rebecca Erschens**

Germany

**Francisco Escobar-Rabadán**

Spain

**Begoña Espejo**

Spain

**Goetz Fabry**

Germany

**Liz Falconer**

UK

**Angela Fan**

Taiwan

**Najat Farsi**

Saudi Arabia

**David Feldstein**

USA

**Li Felländer-Tsai**

Sweden

**Rubens Fernandes**

Brazil

**Laura Fieschi**

Italy

**James Fisher**

UK

**James Fitzgerald**

UK

**Jonathan Flacker**

USA

**Margot Fleuren**

Netherlands

**Maaike Flinkenflögel**

Rwanda

**Signe Flottorp**

Norway

**Cornelia Fluit**

Netherlands

**Eleanor Flynn**

Australia

**Kirsty Foster**

Australia

**Janneke Frambach**

Netherlands

**Chris Frampton**

New Zealand

**Zeno Franco**

USA

**Brandi Freeman**

USA

**Adrian Freeman**

UK

**Garth Funston**

UK

**Daniel Furmedge**

UK

**Sonya Gabrielian**

USA

**Robert Gagnon**

Canada

**Peter Gallagher**

Nigeria

**Jenny Gallagher**

UK

**Gisselle Gallego**

Australia

**Hong Gao**

USA

**Ana Garces**

Guatemala

**Jayne Garner**

UK

**Mark Garside**

UK

**Fasika Amdeslasie Gebrekirkos**

Ethiopia

**David Gijbels**

UK

**Kirsty Gillgrass**

UK

**Ricci Giovanna**

Italy

**Nadine Göb**

Germany

**Joanne Goldman**

Canada

**Kenneth Goldschneider**

USA

**Melody Goodman**

USA

**Adam Gordon**

UK

**Chivaugn Gordon**

South Africa

**Deepthiman Gowda**

USA

**Gertraud Gradl**

Germany

**Jasnet Grant**

UK

**Maria Caterina Grassi**

Italy

**Andy Gray**

South Africa

**Ben Gray**

New Zealand

**Page Gregory**

UK

**Larry Gruppen**

USA

**Anthony Guerrero**

USA

**Bruno Guimarães**

Portugal

**Lisa Guirguis**

Canada

**Freedom Nkhululeko Gumedze**

South Africa

**Ronny Gunnarsson**

Australia

**Geney Gunston**

South Africa

**Puneet Gupta**

India

**Susan Guralnick**

USA

**Michele Haight**

USA

**Iassen Halatchliyski**

Germany

**Suraya Hamid**

Malaysia

**John Hamilton**

Australia

**Dana Hammer**

USA

**Wolfgang Hampe**

Germany

**Heeyoung Han**

USA

**Ailish Hannigan**

Ireland

**Cynthia Haq**

USA

**Eugene Harris**

USA

**Reema Harrison**

Australia

**Christopher Harrison**

UK

**James Harrison**

USA

**Daniel Hashimoto**

USA

**Andrew Hassell**

UK

**Rosemarie Hatala**

Canada

**Kamila Hawthorne**

UK

**Richard Hays**

Australia

**Iman Hegazi**

Australia

**Elizabeth Hemphill**

Australia

**Jeroen Hendriks**

Australia

**Marcus Henning**

New Zealand

**Mark Hernandez**

Ecuador

**Leonie Heskin**

Ireland

**Kathleen Hickey**

USA

**Andrew Hill**

New Zealand

**Ming-Jung Ho**

Taiwan

**Yvonne Hodgson**

Australia

**David Hogan**

Canada

**Are Holen**

Norway

**Daniel Holt**

USA

**Matthias Holzer**

Germany

**Matt Homer**

UK

**Jianlin Hou**

China

**Wendy Hu**

Australia

**Chin-Chou Huang**

Taiwan

**Judith Hudson**

Australia

**Nicky Hudson**

Australia

**Christine Hughes**

Canada

**Daniel Huhn**

Germany

**Steinar Hunskaar**

Norway

**Fawzia Huq**

USA

**Sören Huwendiek**

Switzerland

**Nahla Ibrahim**

Saudi Arabia

**December Ikah**

UK

**Dragan Ilic**

Australia

**Rintaro Imafuku**

Japan

**Kevin Imrie**

Canada

**Gerard Ingham**

Australia

**Farhana Irfan**

Saudi Arabia

**Fabian Jacobs**

Germany

**Mohammad Jalili**

Iran

**Saroj Jayasinghe**

Sri Lanka

**Anthony Jerant**

USA

**Diane Jette**

USA

**Jun Jin**

Hong Kong

**Maree Johnson**

Australia

**Carolyn Johnston**

UK

**Diana Jonas-Dwyer**

Australia

**Michael Jones**

Australia

**Melvyn Jones**

UK

**Therese Jones**

USA

**Christine Jorm**

Australia

**Tanisha Jowsey**

New Zealand

**Noelle Junod Perron**

Switzerland

**Haytham Kaafarani**

USA

**Amudha Kadirvelu**

Malaysia

**Martina Kadmon**

Germany

**Therese Kairuz**

Australia

**Lisa Kane Low**

USA

**Maher Karam**

USA

**Andre Karger**

Germany

**Syed Irfan Karim**

Saudi Arabia

**Indika Karunathilake**

Sri Lanka

**Adetayo Kasim**

UK

**Aliya Kassam**

Canada

**Anne Kawamura**

Canada

**Yang Ke**

China

**Melanie Keep**

Australia

**Carolina Keijsers**

Netherlands

**Michelle Kelly**

Australia

**Martina Kelly**

Canada

**Amanda Kenny**

Australia

**Athol Kent**

South Africa

**Junaid Sarfraz Khan**

Pakistan

**Umatul Khoiriyah**

Indonesia

**Jens Folke Kiilgaard**

Denmark

**James Kilgour**

UK

**Sharla King**

Canada

**Anne Kirchhoff**

USA

**Irene Betty Kizza**

Uganda

**Linda Klein**

Australia

**Robert Kleinert**

Germany

**Petra Knaup**

Germany

**Richard Knox**

UK

**Sarah Koenig**

Germany

**Jennifer Kogan**

USA

**Jillian Kohler**

Canada

**Joseph Kolars**

USA

**Michaela Kolbe**

Switzerland

**Jill Konkin**

Canada

**Andrzej Kononowicz**

Sweden

**Andries Koster**

Netherlands

**Klaus Kroencke**

Germany

**Edward Krupat**

USA

**Kulamakan Kulasegaram**

Canada

**Koshila Kumar**

Australia

**Grace M Kuo**

USA

**Viji Kurup**

USA

**Rashmi Kusurkar**

Netherlands

**Patrick Kyamanywa**

Rwanda

**Anita Laidlaw**

UK

**Trevor Lambert**

UK

**Ekaterini Lambrinou**

Cyprus

**Andrew Lane**

Australia

**Vicki Langendyk**

Australia

**Susan Langmore**

USA

**Marianna Lanoue**

USA

**Philip Larkin**

Ireland

**Jason Last**

Ireland

**Anandi Law**

USA

**Elizabeth Lazzara**

USA

**Heather Leary**

USA

**Michael Leathley**

UK

**Nikki Lee**

Netherlands

**Wui-Chiang Lee**

Taiwan

**Jennifer Leeman**

USA

**Janet Lefroy**

UK

**Ronny Lehmann**

Germany

**Paolo Leombruni**

Italy

**Jimmie Leppink**

Netherlands

**Kat Leung**

Australia

**Linda Lewin**

USA

**Robin Lewis**

UK

**Su-Ting Li**

USA

**Sok Ying Liaw**

Singapore

**Steven Lillis**

New Zealand

**Blossom Lin**

Taiwan

**Michelle Lincoln**

Australia

**Stefan Lindgren**

Sweden

**Liliane Lins**

Brazil

**Kristin Lo**

Australia

**Jocelyn Lockyer**

Canada

**Stephen Loftus**

USA

**Lia Logio**

USA

**Lawrence Loo**

USA

**Elizabete Loureiro**

Portugal

**Alwyn Louw**

South Africa

**Karen Luetsch**

Australia

**Robert Michael Lundin**

UK

**Paul Lyons**

USA

**Monica Lypson**

USA

**Marion Maar**

Canada

**Martin Macdowell**

USA

**Mohi Eldin Magzoub**

Egypt

**Takatoshi Makino**

Japan

**Marianne Mak-van der Vossen**

Netherlands

**Bunmi Malau-Aduli**

Australia

**Neha Malhotra**

New Zealand

**Stephen Maloney**

Australia

**Silvia Mamede**

Netherlands

**Kate Mandeville**

UK

**Dianne Manning**

South Africa

**Katherine Margo**

USA

**Maurice Mars**

South Africa

**Aaron Marshall**

USA

**Lene Martin**

Sweden

**Yousef Marwan**

Kuwait

**Robert Mash**

South Africa

**Italo Masiello**

Sweden

**David Matheson**

UK

**Edward Matsumoto**

Canada

**Nikos Mattheos**

Hong Kong

**Maliza Mawardi**

Malaysia

**Simon Maxwell**

UK

**Win May**

USA

**Miguel Angel Mayer**

Spain

**Odette Mazel**

Australia

**Ruth McMenamin**

Ireland

**Lynn McBain**

New Zealand

**Lise McCoy**

USA

**Matthew McGrail**

Australia

**Deirdre McGrath**

UK

**Danette McKinley**

USA

**Michelle McLean**

Australia

**Chris McManus**

UK

**Onno Meijer**

Netherlands

**Ryan Meili**

Canada

**Stewart Mennin**

USA

**Sultan Ayoub Meo**

Saudi Arabia

**Annette Mercer**

Australia

**Jacqueline Merrill**

USA

**Neil Merrylees**

UK

**Jamie Meuser**

Canada

**Ricarda Mewes**

Germany

**Barret Michalec**

USA

**Sandy Middleton**

Australia

**Brennen Mills**

Australia

**Taro Minami**

USA

**Logan Mitchell**

New Zealand

**Peter Mittwede**

USA

**Tobias Moczko**

Germany

**Lise Mogensen**

Australia

**Mpho Mogodi**

Botswana

**Victor Mogre**

Ghana

**Simin Mohebbi**

Iran

**Andreas Möltner**

Germany

**Shabir Moosa**

South Africa

**Meghan Moran**

USA

**Anne Mette Morcke**

Denmark

**Prue Morgan**

Australia

**Priya Morjaria**

UK

**Christopher P Morley**

USA

**Alison Mostyn**

UK

**Christine Moutier**

USA

**Phillip Mucksavage**

USA

**Brigitte Mueller-Hilke**

Germany

**Judy Mullan**

Australia

**Ellen Murgitroyd**

UK

**Douglas Murphy**

UK

**Richard Murray**

Australia

**Deirdre Murray**

Ireland

**David Musick**

USA

**Eduardo Mutto**

Argentina

**Erisa Mwaka**

Uganda

**Sun Jung Myung**

South Korea

**Balakrishnan Nair**

Australia

**Devaki Nambiar**

India

**Susan Nancarrow**

Australia

**Abedini Nauzley**

USA

**Hamde Nazar**

UK

**David Ndetei**

Kenya

**Maarit Nevalainen**

Finland

**Anne Nevgi**

Finland

**Jonathan Newbury**

Australia

**Khuen Yen Ng**

Malaysia

**Sandra Nicholson**

UK

**Christoph Nikendei**

Germany

**Kirstin Nillesen**

Netherlands

**Somashekhar Nimbalkar**

India

**Gillian Nisbet**

Australia

**Lorraine Noble**

UK

**Jonas Nordquist**

Sweden

**Birgitte Nørgaard**

Denmark

**Ross Norman**

Canada

**Zineb Nouns**

Switzerland

**Bridget Obrien**

USA

**Sabine Oertelt-Prigione**

Germany

**Bodil Ohlsson**

Sweden

**E Oluwabunmi Olapade-Olaopa**

Nigeria

**Patricia Oliveira**

Brazil

**Asela Olupeliyawa**

Sri Lanka

**Hirotaka Onishi**

Japan

**Amy Opalek**

USA

**Nancy Oriol**

USA

**Helen O'Sullivan**

UK

**Anna Oswald**

Canada

**Colm O'Tuathaigh**

Ireland

**Odile Ouwe Missi Oukem-Boyer**

Cameroon

**Claudio Owino**

Kenya

**Gozde Ozakinci**

UK

**Jorge Pales**

Spain

**Alvisa Palese**

Italy

**Edward Palmer**

Australia

**Mariusz Panczyk**

Poland

**Andrew Papanikitas**

UK

**Yoon Soo Park**

USA

**Helena Paro**

Brazil

**Janet Parsons**

Canada

**Vimmi Passi**

UK

**Joanne Pattinson**

UK

**Andrew Timothy Pattison**

UK

**David Paul**

Australia

**Karl Payne**

UK

**Ian Pearce**

UK

**Jacob Pearce**

Australia

**Jon Penm**

USA

**Pamela Pennington**

Guatemala

**Jason Perepelkin**

Canada

**Adam Persky**

USA

**John Peteet**

USA

**Harm Peters**

Germany

**Ray Peterson**

Australia

**Robin Pettit**

USA

**Julie Phillips**

USA

**Jobeth Pilcher**

USA

**Ralph Pinnock**

New Zealand

**Jason Pole**

Canada

**Susan Pollart**

USA

**David Ponka**

Canada

**Sari Ponzer**

Sweden

**Phillippa Poole**

New Zealand

**Cornelis Postma**

Netherlands

**Cheryl Poth**

Canada

**Merce Prat-Sala**

UK

**Gill Price**

UK

**Titi Savitri Prihatiningsih**

Indonesia

**Amy Prunuske**

USA

**Ian Puddey**

Australia

**Livia Puljak**

Croatia

**Martin Pusic**

USA

**Raghunath Puttaiah**

USA

**Louise Racine**

Canada

**Jan Radford**

Australia

**Natalie Radomski**

Australia

**Malathi Raghavan**

Canada

**Yinusa Raji**

Nigeria

**Vijaykumar Rajput**

USA

**Christopher Ramnanan**

Canada

**Fiza Rashid-Doubell**

Bahrain

**Tobias Raupach**

Germany

**Robin Ray**

Australia

**Mark Raymond**

USA

**Szabo Rebecca**

Australia

**John Rees**

UK

**Paul Rega**

USA

**Krishna Regmi**

UK

**Rehana Rehman**

Pakistan

**Kate Reid**

Australia

**Marcel Reinders**

Netherlands

**Shmuel Reis**

Israel

**L Reis**

Brazil

**Daniel Reissmann**

Germany

**Harold Reiter**

Canada

**Jeremy Richards**

USA

**Janet Riddle**

USA

**Arnoldo Riquelme**

Chile

**Christopher Roberts**

Australia

**Katie Robinson**

USA

**Kem Rogers**

Canada

**Marcy Rosenbaum**

USA

**Tara Rosewall**

Canada

**Shelley Ross**

Canada

**Linda Ross**

Australia

**Jens Rothenberger**

Switzerland

**Imogene Rothnie**

Australia

**Charlotte Rothwell**

UK

**Joy Rudland**

New Zealand

**Miriam Rüsseler**

Germany

**Clark Russell**

UK

**Aline Saad**

Lebanon

**Hazim Sadideen**

UK

**Ratana Saipanish**

Thailand

**Eduardo Salas**

USA

**Helen Salisbury**

UK

**Murali Sambasivan**

Malaysia

**Dario Sambunjak**

Croatia

**Sally Santen**

USA

**Marina Sawdon**

UK

**Ana Sergio Da Silva**

UK

**Stefan K Schauber**

Germany

**Noah Schenkman**

USA

**Sarah Schiekirka**

Germany

**Carolin Schmid**

Germany

**Ralf Schmidmaier**

Germany

**Kai Philipp Schnabel**

Switzerland

**Francine Schneider**

Netherlands

**Andreas Schröder**

Denmark

**Anne-Mette Schroll**

Denmark

**Jobst-Hendrik Schultz**

Germany

**Daniel Schumacher**

USA

**Katrin Schüttpelz-Brauns**

Germany

**Karen Scott**

USA

**Jane Seale**

UK

**Dean Seehusen**

USA

**Ahsan Sethi**

UK

**Saran Shantikumar**

UK

**John Shaw**

New Zealand

**Dale Sheehan**

New Zealand

**Emma Sherry**

Australia

**Duncan Shrewsbury**

UK

**Boaz Shulruf**

Australia

**Matt Sibbald**

Canada

**D Robert Siemens**

Canada

**Si Mui Sim**

Malaysia

**Anne Simmenroth-Nayda**

Germany

**Christopher Simon**

Canada

**Gaiane Simonia**

Georgia

**Scot Simpson**

Canada

**Vicki Simpson**

USA

**Veena Singaram**

South Africa

**Kuldeep Singh**

Nepal

**Katrin Singler**

Germany

**Tony Skapetis**

Australia

**Ruth Sladek**

Australia

**Helen Slater**

Australia

**Lars Småbrekke**

Norway

**Sydney Smee**

Canada

**Claire Smith**

UK

**Robert Smith**

USA

**Stefanus Snyman**

South Africa

**Ingunn Bjarnadottir Solberg**

Norway

**Zdenko Sonicki**

Croatia

**Penny Standen**

USA

**Karen Stegers-Jager**

Netherlands

**Jost Steinhäuser**

Germany

**Aslak Steinsbekk**

Norway

**Fred Stevens**

Netherlands

**Michael Stevenson**

UK

**Lyn Stewart**

Australia

**Rosalyn Walker Stewart**

USA

**Fredrich Stiefel**

Switzerland

**Philipp Stieger**

Germany

**Alasdair Strachan**

UK

**Judith Strawbridge**

Ireland

**Goran Strkalj**

Australia

**Alison Sturrock**

UK

**Virpi Sulosaari**

Finland

**Samantha Sundercombe**

Australia

**Tarja Suominen**

Finland

**Premala Sureshkumar**

Australia

**Laura Surmon**

Australia

**Jeannette L Swaan**

Netherlands

**Meenakshi Swamy**

UK

**Nur Afrainin Syah**

Indonesia

**Shima Tabatabai**

Iran

**Masami Tagawa**

Japan

**Kenzo Takahashi**

Japan

**Zohray Talib**

USA

**Marie Tarrant**

Hong Kong

**Ara Tekian**

USA

**Conor Teljeur**

Ireland

**Olle ten Cate**

Netherlands

**Pim Teunissen**

Netherlands

**Nai-Peng Tey**

Malaysia

**Axel Themmen**

Netherlands

**Kassiani Theodoraki**

Greece

**Johan Thor**

Sweden

**Sean Tierney**

Ireland

**Paul Tiffin**

UK

**Julie K Tilson**

USA

**Vivienne Tippett**

Australia

**Jonathon Tomlinson**

UK

**Jane Toms**

UK

**William Tormey**

Ireland

**Deborah Tregunno**

Canada

**Marc Triola**

USA

**Shih-Li Tsai**

Taiwan

**Ilene Tsui**

USA

**Sharon Tucker**

USA

**Mike Tweed**

New Zealand

**Reidar Tyssen**

Norway

**Jinan Usta**

Lebanon

**Sjoukje van den Broek**

Netherlands

**Cees van der Vleuten**

Netherlands

**Nynke van Dijk**

Netherlands

**Tamara van Gog**

Netherlands

**Clare van Hamel**

UK

**Daniël van Nijlen**

Belgium

**Dirk van Rooy**

Australia

**Gerrit van Schalkwyk**

USA

**Susan van Schalkwyk**

South Africa

**Chris van Weel**

Australia

**Marta van Zanten**

USA

**Cristina Vargas**

Spain

**Gary Velan**

Australia

**Joan Vilalta-Franch**

Spain

**Laura Villa-Torres**

USA

**Claudio Violato**

USA Minor Outlying Islands

**Michelle Visser**

USA

**Elsa Vitale**

Italy

**Pirashanthie Vivekananda-Schmidt**

UK

**Carl von Baeyer**

Canada

**Rukhsana W Zuberi**

Pakistan

**Judy Wakeling**

UK

**Gary Walco**

USA

**Rachel Walker**

Australia

**Merrilyn Walton**

Australia

**Jennifer Walton**

Canada

**Helena Ward**

Australia

**Terri Warholak**

USA

**Justin Waring**

UK

**Simon Watmough**

UK

**Andy Wearn**

New Zealand

**Katie Webb**

UK

**Jennifer Weller**

New Zealand

**Olwyn Westwood**

UK

**Sue Whittle**

UK

**Kristin Wiisanen Weitzel**

USA

**Marjo Wijnen-Meijer**

Netherlands

**Deana Wilbanks**

USA

**Kerry Wilbur**

Qatar

**Christine Wilder**

USA

**Margaretha Wilhelmsson**

Sweden

**Jill Wilkinson**

New Zealand

**Ingrid Willaing**

Denmark

**Lawrence Williams**

USA

**Graham Williamson**

UK

**Kathleen Williamson**

USA

**Deanna Willis**

USA

**Ian Wilson**

Australia

**Adam Wilson**

USA

**Khin Than Win**

Australia

**Abigail Winkel**

USA

**Erin Winstanley**

USA

**Connie Wiskin**

UK

**Ulrich Woermann**

Switzerland

**Zane Wolf**

USA

**Yut Lin Wong**

Malaysia

**Raymond Wong**

USA

**Timothy Wood**

USA

**Robyn Woodward-Kron**

Australia

**Bjarne Worm**

Denmark

**Kei Yamada**

USA

**Che-Ming Yang**

Taiwan

**Yanqi Yang**

Hong Kong

**Ahmed Yaqinuddin**

Pakistan

**Kwang Chien Yee**

Australia

**Liuhua Ying**

China

**James Youdas**

USA

**Greg Young**

USA

**Omar Young**

USA

**Tzu-Chieh Yu**

New Zealand

**Steven Yule**

USA

**Zamros Yusof**

Malaysia

**Nikki Zaidi**

USA

**Benjamin Zendejas**

USA

**Melvyn Zhang**

Singapore

**Zhao-Hua Zhong**

UK

**Stefan Zimmermann**

Germany

**Sa'ed Zyoud**

Palestinian Territory

**Aleksander Zywot**

Grenada

